# Enhancing heart and circulatory well-being through optimized radial artery techniques: a meta-analysis of hemostasis and patient comfort

**DOI:** 10.3389/fcvm.2024.1412479

**Published:** 2025-01-15

**Authors:** Yanru Yang, Hongyan Zhu, Guangyao Zhai

**Affiliations:** Department of Cardiovascular Medicine, Capital Medical University, Beijing LuHe Hospital, Beijing, China

**Keywords:** radial artery hematoma radial artery compression time, radial artery occlusion, bleeding complications, patient comfort radial access, transradial band device

## Abstract

**Objective:**

This meta-analysis elucidates the efficacy of the Transradial Band Device (TR Band) in minimizing complications like radial artery occlusion and hematoma, preserving heart health, and enhancing blood flow post-transradial catheterization.

**Methods:**

A comprehensive literature search across databases including PubMed, Cochrane, and Embase examined the impact of radial artery compression techniques and decompression times on complications. Data from 13 studies were analyzed using R 4.1.2 with fixed-effects and random-effects models. The Newcastle-Ottawa Scale assessed the risk of bias in observational cohort studies.

**Results:**

In our meta-analysis, we evaluated data from various studies encompassing different air volumes in transradial band devices across several outcomes including bleeding, hematoma, radial artery occlusion (RAO), Visual Analog Scale (VAS) scores, and compression time. The collective analysis integrated findings from 11 studies, totaling 4,679 patients. No significant difference in bleeding risk (OR 1.04, 95% CI 0.60–1.82, *p* > 0.05, *I*^2^ = 78%), hematoma incidence (OR 0.96, 95% CI 0.78–1.19, *p* > 0.05, *I*^2^ = 0%), or RAO incidence (OR 0.96, 95% CI 0.78–1.19, *p* > 0.05, *I*^2^ = 0%) was observed between the “Less air” and “15 ml air” groups. However, the “Less air” group reported significantly higher VAS scores indicating increased pain or discomfort (Mean Difference 0.25, 95% CI 0.09–0.41, *p* < 0.05, *I*^2^ = 0%). Compression time analyses showed no significant difference between groups (Mean Difference −17.73, 95% CI −54.65–19.20, *p* > 0.05, *I*^2^ = 99%). Sensitivity analyses confirmed the stability of these findings, and Egger's test indicated no significant publication bias across the outcomes. This synthesis highlights the nuanced impact of air volume adjustments in transradial bands on patient outcomes, emphasizing the necessity for further research and standardized protocols to optimize patient safety and comfort post-intervention.

**Conclusion:**

The TR Band, when utilized with optimized air volume/pressure, maintains an essential balance between ensuring hemostasis and enhancing patient comfort without elevating the risk of radial artery complications. These findings support the careful selection of TR Band settings to optimize clinical outcomes in patients undergoing transradial cardiac procedures. Further research is warranted to establish standardized guidelines for the most effective use of TR Band in various clinical scenarios.

## 1 Introduction

The emergence of transradial access (TRA) for cardiac catheterizations and percutaneous coronary interventions has markedly altered the practice of cardiovascular medicine, presenting a preferential alternative to the conventional transfemoral approach. Research indicates that TRA not only minimizes the risk of bleeding complications but also enhances patient comfort and accelerates recovery times, leading to its increasing adoption among healthcare professionals (1, 2). Despite its numerous benefits, TRA is not without potential drawbacks, including the risks of radial artery occlusion (RAO) and hematoma, which may compromise the integrity of arterial pathways, restrict future vascular access options, and occasionally necessitate surgical correction (3–5).

The development and utilization of the Transradial Band Device (TR Band) have been identified as crucial advancements aimed at reducing these complications. By applying controlled compression at the radial artery puncture site, the TR Band aids in achieving hemostasis while simultaneously lowering the risk of arterial occlusion and hematoma (6). Nonetheless, determining the optimal application parameters for the TR Band, including the exact air volume/pressure and compression duration necessary to balance hemostasis with minimal risk of complications, continues to be a focal point of research and discussion within the medical community (7–9).

This meta-analysis examines the vital role of the TR Band in enhancing cardiac health and blood flow following transradial procedures by systematically reviewing available literature on its effectiveness in reducing RAO and hematoma occurrences. It further explores the impact of varying TR Band application techniques on patient comfort and procedural outcomes, offering valuable insights into establishing best practices for its use. Through this examination, the study aims to contribute significantly to the ongoing conversation regarding the optimal employment of the TR Band in TRA procedures, highlighting its potential to not only safeguard procedural integrity but also improve the overall patient experience. This research underscores the imperative of advancing transradial methodologies and technologies to continue the progression of interventional cardiology.

## 2 Methodology

### 2.1 Search strategy

**Figure 1:**
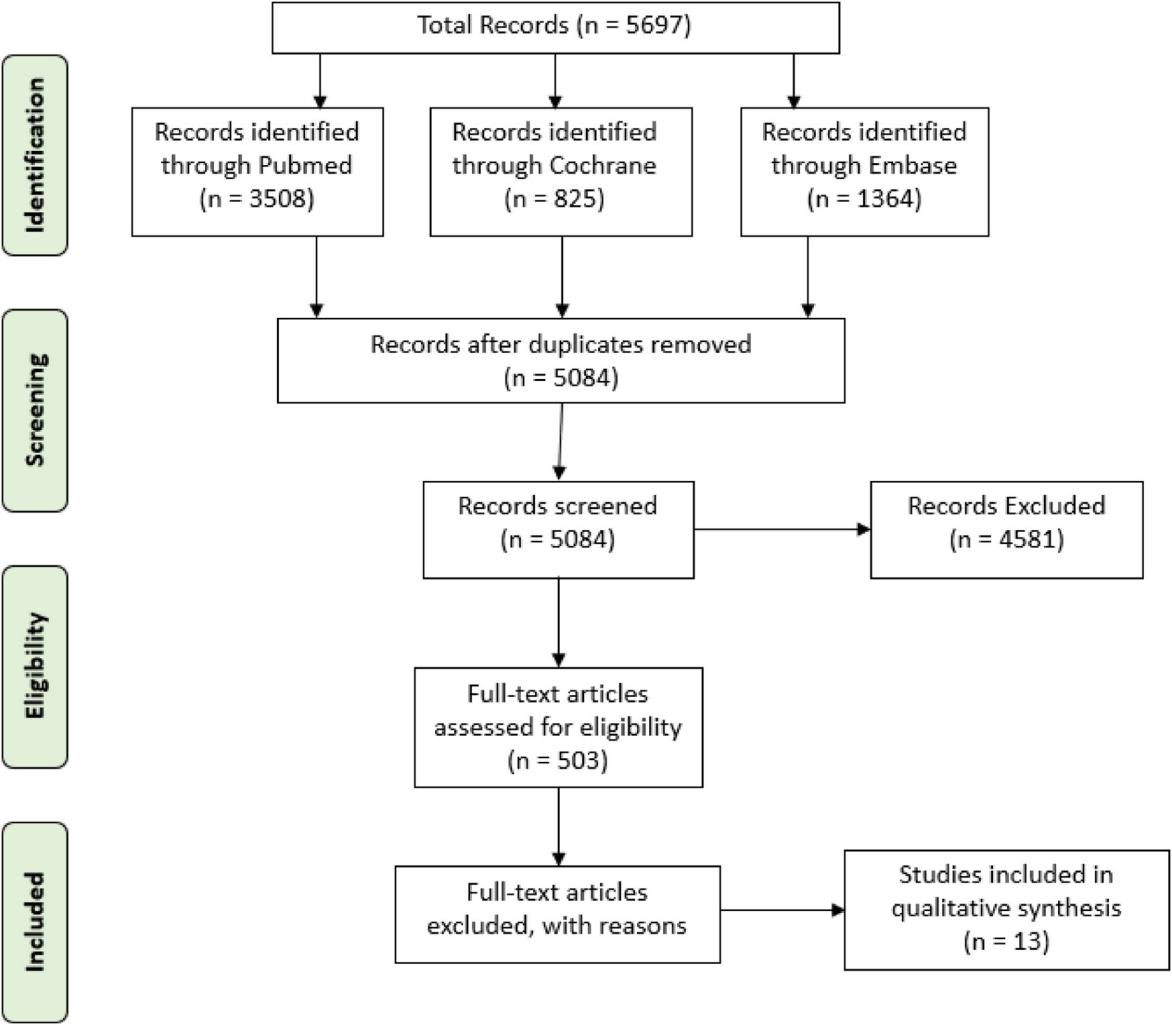
PRISMA flow diagram illustrating the search strategy. This flow diagram provides the phases of article identification and selection, which resulted in the identification of 13 articles that were deemed eligible for inclusion in the review.

To compile a robust dataset for this meta-analysis, an extensive literature search was conducted across multiple databases, focusing on the efficacy of various interventions in preventing TRA complications. The databases searched included PubMed, Cochrane Library, and Embase, utilizing specific keywords and combinations such as “Transradial” and “Transradial AND pressure” or “Transradial AND blood”. This search strategy aimed to capture a broad spectrum of studies relevant to TRA complications and interventions, particularly focusing on the application pressures and decompression times associated with the TR Band. The search yielded a total of 3,508 papers from PubMed (with 2 duplicates), 825 trials from Cochrane (with 1 duplicate), and 1,364 papers from Embase (with 610 duplicates), indicating a significant interest and body of research in this area (PubMed, 2021; Cochrane, 2021; Embase, 2021). The PRISMA diagram, as shown in Figure 1, details the stages of identifying and selecting papers.

### 2.2 Statistical analysis

The meta-analysis was conducted using R version 4.1.2, a statistical software known for its robustness in handling complex data analyses. The primary outcomes, such as bleeding, hematoma formation, and radial artery occlusion (RAO), were summarized as weighted mean differences (WMD) of continuous variables with 95% confidence intervals (CIs). This analysis utilized both fixed-effects and random-effects models with inverse variance weighting to account for the variability among studies. Heterogeneity among studies was evaluated using the *I*^2^ test, categorizing it as low (*I*^2^ < 25%), moderate (25% ≤ *I*^2^ ≤ 50%), or substantial (*I*^2^ > 50%). To assess the risk of bias in the included observational cohort studies, the Newcastle-Ottawa Scale was applied, ensuring the reliability and validity of the findings (10).

### 2.3 Studies characteristics and outcomes

The studies included in this meta-analysis varied in their design, sample size, and the interventions tested. These ranged from alternative compression devices and manual techniques to the standardized use of the TR Band with specific air volumes/pressures. Notably, comparisons were made between groups receiving less air volume/pressure and those with the standard 15 ml air volume/pressure in the TR Band, examining outcomes such as bleeding, hematoma, RAO, and patient-reported visual analogue scale (VAS) scores for pain/discomfort. The characteristics and outcomes of these studies highlight the diversity of approaches in managing TRA complications and underscore the importance of optimizing TR Band application for improved patient care.

## 3 Results

### 3.1 Studies characteristics

Specifically focusing on RAO, contrasting methods such as conventional pressure dressing and sham PO-FMD were evaluated for their preventative efficacy, contributing valuable data to the ongoing discourse on optimal post-procedural care (11, 12). The exploration of the effectiveness of chitosan-based pads, elastic bandages, and varying air volumes in TR bands in studies addressed a range of outcomes from bleeding to compression time, highlighting the importance of device selection and procedural nuances (13, 14). Notably, the utilization of manual compression vs. mechanical compression in a significant cohort presented a pivotal analysis on the comparative benefits of each method, further enriching the understanding of best practices in minimizing complications (15). Additionally, an innovative approach involving patent hemostasis compared with traditional methods offered a unique perspective on enhancing patient outcomes and safety post-intervention (16). Collectively, these studies (11–24) underscore the nuanced implications of transradial compression techniques on patient safety and comfort, advocating for continued research to establish more refined and standardized care protocols. Table 1, provides an exhaustive comparison of the outcomes from using varying air pressures in transradial band devices, illustrated through a selection of studies.

**Table 1:** Comparative analysis of intervention efficacy using different Air pressures in transradial band devices across Various studies.

	Studies	Less air (other compress devices)	15 ml air (TR band)	Air volume/pressure of Less air	Air volume/pressure of 15 ml air	No. of less air	No. of 15 ml air	Outcomes	Selection _NOS	Comparability _NOS	Outcome _NOS	Nos
1	Bardooli 2023	AIR band	TR band	7 ml air	13–15 ml air	50	50	Bleeding, RAO	3	2	1	6
2	Due 2021	RY stop	TR band	Padded plastic support stretching, no air	18 ml air	248	251	Bleeding, hematoma, RAO, VAS	4	2	3	9
3	Cong 2016	Pressure dressing	TR band	–	–	550	550	Bleeding, hematoma, RAO, VAS	4	2	3	9
4	Cubero 2009	TR band	TR band	Mean artery pressure, 8.8 ± 1.7 ml air	15 ml air	176	175	Bleeding, hematoma, RAO, compression time	4	2	3	9
5	Dai 2015	Chitosan-based pad	TR band	Elastic bandage, no air	8 + 8 ml air	300	300	Bleeding, RAO, compression time	4	2	3	9
6	Dangoisse 2017	TR band	TR band	10 ml air	13 ml air	1246	691	Bleeding, hematoma, RAO, compression time	4	2	3	9
7	Dos santos 2020	Conventional pressure dressing	TR band	Folded gauze + elastic adhesive bandages, no air	15 ml air	299	301	RAO	4	2	3	9
8	Doubell 2021	Sham PO-FMD	PO-FMD	Blood pressure cuff without air	Blood pressure cuff with air	570	560	RAO	4	2	3	9
9	Kang 2017	Compression device and a chitosan-based pad	Compression devices alone	–	–	59	36	Bleeding, hematoma	4	2	3	9
10	Petroglou 2018	Manual compression	Mechanical compression	–	–	304	285	Bleeding, hematoma, RAO	4	2	3	9
11	Roghani 2017	Patent hemostasis	Traditional hemostasis	10 ml air	12 ml air	60	60	Bleeding, hematoma, RAO, compression time	4	1	3	8
12	Wang 2018	New hemostatic compression device	TR band	Air until 250 mmHg	14–16 ml	59	59	Hematoma, RAO	4	2	3	9

### 3.2 Bleeding

#### 3.2.1 Bleeding risk with varied TR band air pressure

**Figure 2:**
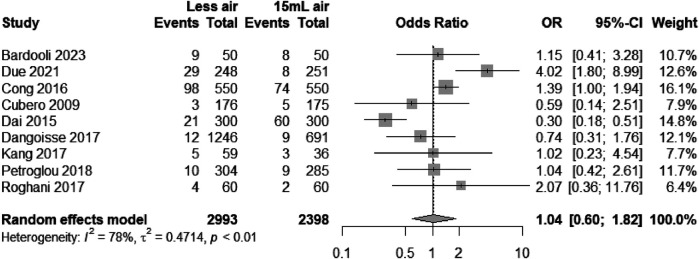
Odds ratios for bleeding with “Less air” vs. “15 ml air” in TR bands, showing no significant difference.

The forest plot in Figure 2 synthesizes data from multiple studies to compare the risk of bleeding between groups using transradial bands with different air volumes. The aggregate odds ratio (OR) of 1.04 with a 95% confidence interval (CI) from 0.60 to 1.82 crosses the null effect line (OR = 1), indicating no significant difference in bleeding risk between the “Less air” and “15 ml air” groups (11, 12, 15, 16, 18–21, 23). Nonetheless, the considerable heterogeneity (*I*^2^ = 78%) suggests variation across studies, which may reflect differences in methodologies or patient populations. This heterogeneity warrants a careful interpretation of the results and highlights the need for standardized definitions and protocols in future research to clarify the influence of air volume on bleeding outcomes following transradial interventions. The combined results show no significant difference in bleeding frequency, with substantial heterogeneity across studies.

#### 3.2.2 Robustness of bleeding risk analysis with TR band air pressure variability

**Figure 3:**
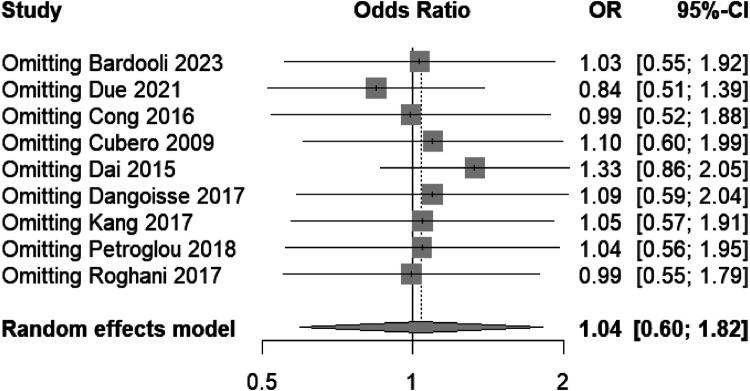
Sensitivity analysis indicating consistent bleeding risk across studies, affirming the meta-analysis robustness.

The forest plot (Figure 3) appears to be a sensitivity analysis examining the effect of omitting each individual study on the pooled odds ratio of bleeding events when using transradial band devices. The plot shows the recalculated pooled odds ratios excluding each study in turn. Each row specifies the study that was omitted and the resulting odds ratio with its 95% confidence interval (CI). The squares represent the recalculated odds ratio when each study is omitted, and the horizontal lines through the squares represent the 95% CI. The diamond represents the overall pooled odds ratio based on the random-effects model when all studies are included.

The overall pooled odds ratio remains stable and not significantly different from 1 (no effect), as evidenced by the diamond's center remaining close to the line of no effect (OR = 1). This suggests that no single study disproportionately influences the meta-analysis outcome, indicating a robust result across the studies included.

**Figure 4:**
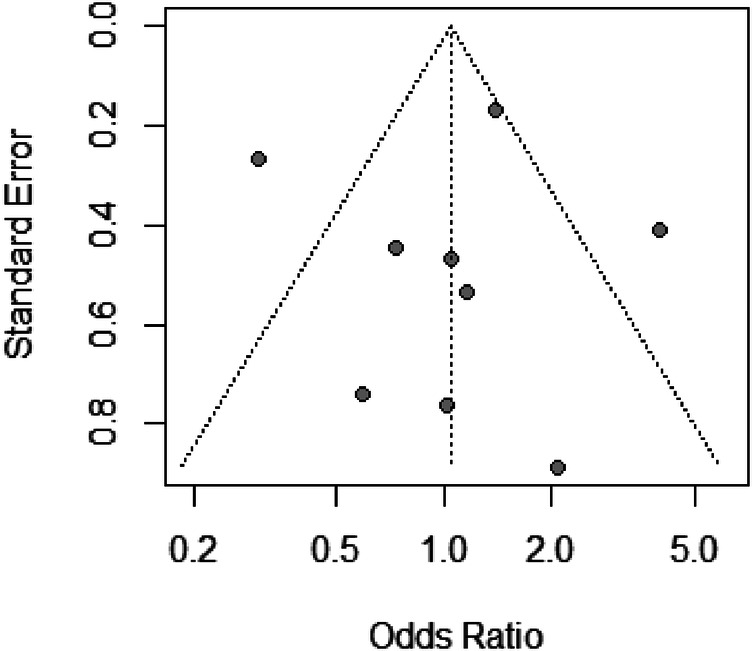
Funnel plot of standard error by odds ratio for bleeding risk studies, indicating potential publication bias.

The funnel plot (Figure 4) displays a symmetrical distribution around the aggregate odds ratio, suggesting no apparent publication bias in the studies examining bleeding risks with different air volumes in transradial band devices. This symmetry, coupled with the sensitivity analysis showing minimal fluctuation in odds ratios when individual studies are omitted, indicates robust and unbiased results in our meta-analysis.

In our analysis, Egger's test yields a *t*-value of 0.02 with 7° of freedom, resulting in a *p*-value of 0.9811. This high *p*-value suggests that there is no significant asymmetry within the funnel plot, and hence, no substantial evidence of publication bias. The estimated bias is minimal (0.0391) with a standard error of bias (se.bias) of 1.5961, and an intercept (0.0175) close to zero with its standard error (se.intercept) of 0.5337, further indicating no notable bias in the analysis. The multiplicative residual heterogeneity variance (tau^2^ = 5.7805) reflects variability among the true effects in the analyzed studies, which is accounted for in the model. The use of standard error as a predictor and the inverse variance of score as a weight in the regression model is standard practice in Egger's test, following the methodological approach outlined by Harbord et al. (25) in “Statistical Methods in Medical Research”. This statistical result supports the visual interpretation of the funnel plot, corroborating the robustness of our meta-analysis findings regarding bleeding risks with different air volumes in transradial band devices.

### 3.3 Hematoma

#### 3.3.1 Hematoma incidence in transradial interventions with varying air volumes

**Figure 5:**
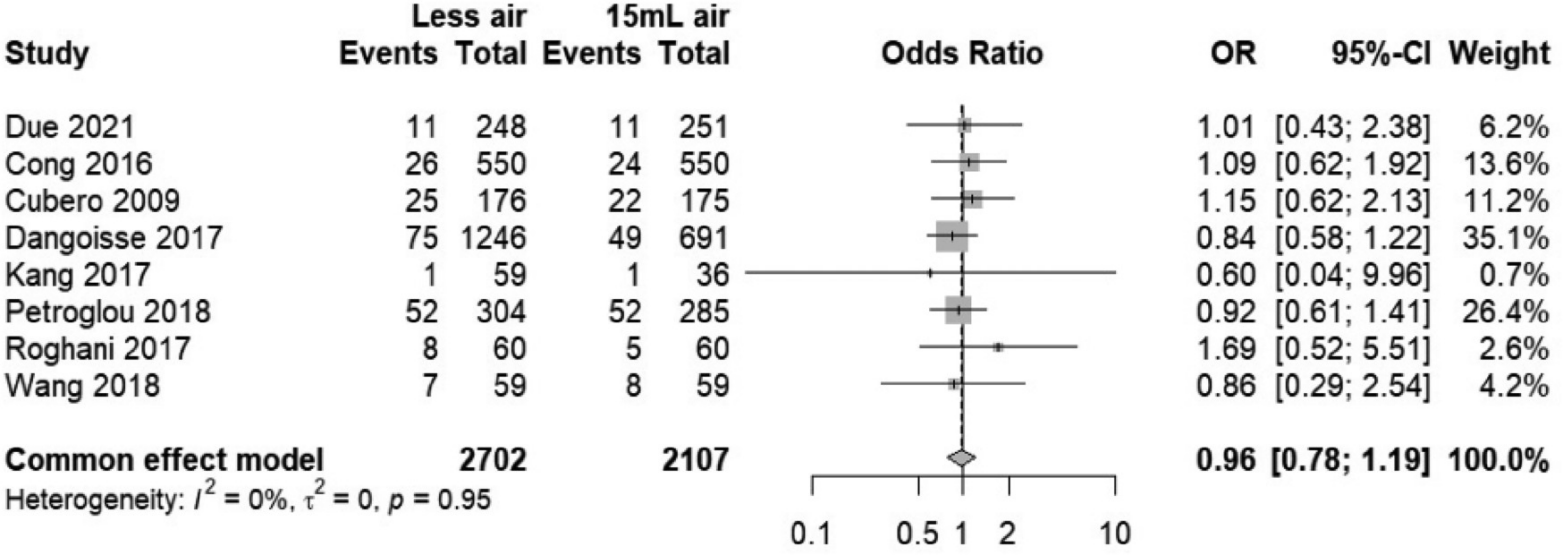
Forest plot analysis of hematoma events, comparing “Less air” versus “15 ml air” groups.

The forest plot (Figure 5) visualizes the comparison of hematoma incidence between “Less air” and “15 ml air” groups across several studies (3, 12, 15, 16, 18, 20, 21, 23, 24). With a combined odds ratio of 0.96 and a 95% confidence interval of 0.78–1.19, the analysis indicates no statistically significant difference in hematoma frequency between the two groups. The lack of heterogeneity (*I*^2^ = 0%, τ^2^ = 0, *p* = 0.95) suggests that the results are consistent across studies, strengthening the evidence that variations in air volume within the transradial band devices do not influence the risk of hematoma. This homogeneity of effect sizes and the narrow confidence intervals around an odds ratio of 1 imply a high level of precision and reliability in the reported outcomes.

#### 3.3.2 Sensitivity analysis for hematoma risk in transradial band studies

**Figure 6:**
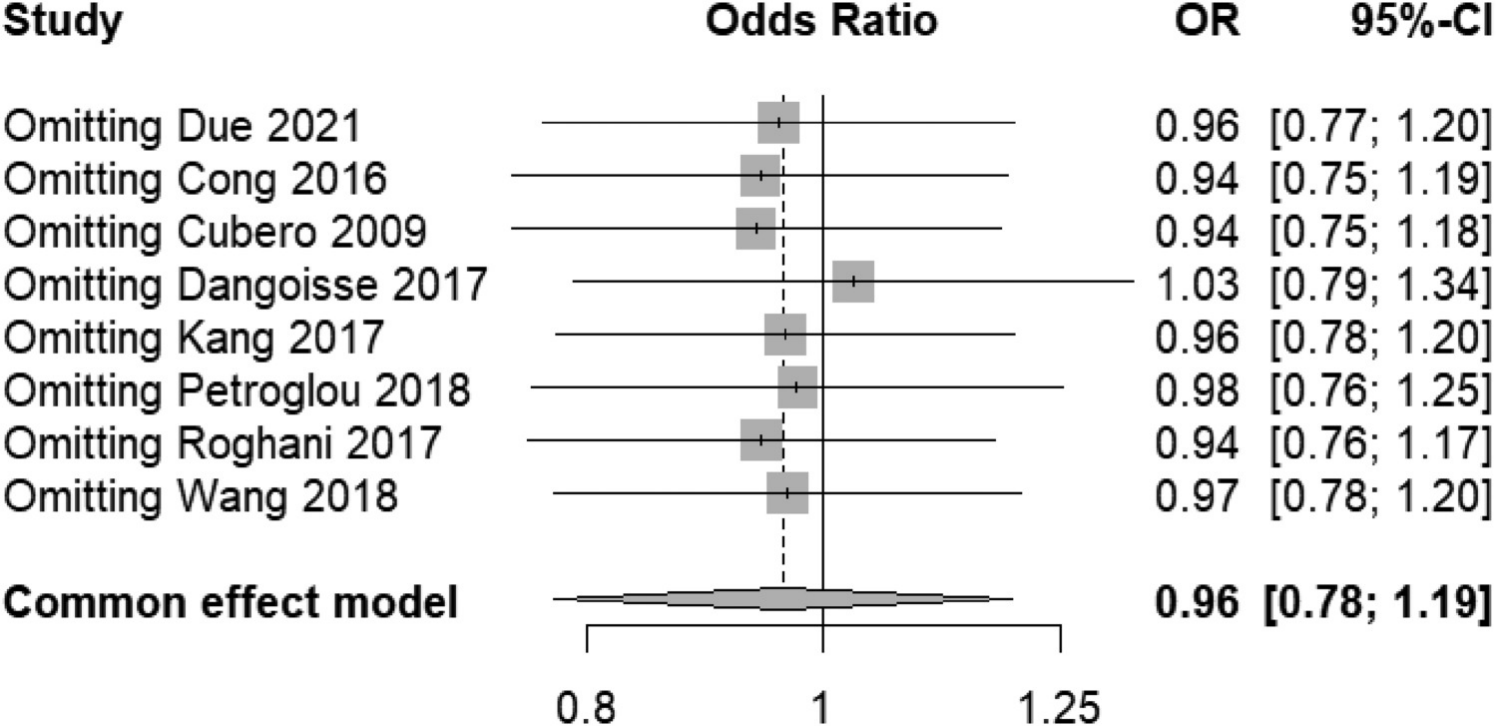
Sensitivity analysis forest plot for hematoma risk, demonstrating stability across studies.

This sensitivity analysis forest plot (Figure 6) evaluates the influence of individual studies on the overall effect size concerning hematoma risk in transradial band studies. The plot illustrates that the omission of any single study does not significantly change the combined odds ratio, which remains close to 1.0 (0.96), with narrow 95% confidence intervals consistently spanning across the line of no effect. This suggests that the meta-analysis results are robust and not dependent on any single study, providing confidence in the conclusion that the difference in air volume within transradial band devices does not significantly affect hematoma risk. The consistency of these findings, with minimal variation in effect sizes upon the removal of individual studies, underscores the reliability of the reported outcomes.

**Figure 7:**
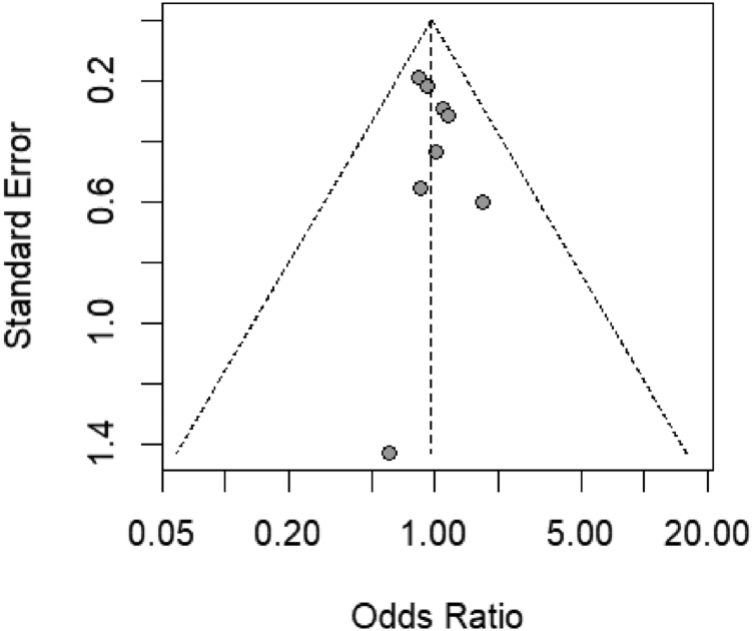
Funnel plot assessing publication bias in studies comparing hematoma risk with varying TR band air volume.

The funnel plot in Figure 7, when coupled with the results of Egger's test, provides a statistical assessment of publication bias in our meta-analysis examining the risk of hematoma associated with different air volumes in transradial band devices. The symmetry of the plot, with studies distributed on both sides of the aggregate odds ratio, suggests a low likelihood of publication bias, which is statistically supported by Egger's test yielding a t-value of 0.88 (df = 6) and a *p*-value of 0.4150. This *p*-value, being greater than the conventional alpha level of 0.05, indicates no significant asymmetry and hence no substantial evidence of publication bias. The sample estimates showing a slight bias with a small standard error further support the lack of significant skew in the distribution of studies. The residual heterogeneity variance (tau^2^ = 0.3129) within the analysis is modest, indicating that the variance between the true effects of the studies is not excessively large. This evaluation, rooted in the methodology of Harbord et al. (2006), reinforces the reliability of the conclusion that air volume differences in TR bands do not significantly impact hematoma risks.

### 3.4 Radial artery occlusion (RAO)

#### 3.4.1 Assessment of radial artery occlusion risks associated with TR band air volumes

**Figure 8:**
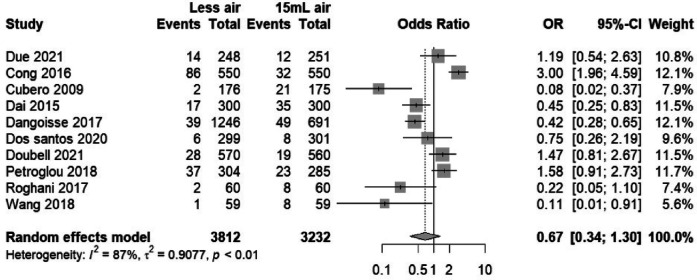
Forest plot showing RAO odds ratios for “Less air” versus “15 ml air” groups.

The forest plot (Figure 8) presents a meta-analysis of the odds ratios (OR) for radial artery occlusion (RAO) incidents across various studies (11–14, 16, 18, 20, 21, 23, 24), comparing two intervention groups defined by the air volume in transradial band devices. The plot demonstrates that the pooled OR is less than 1 (OR = 0.96), suggesting a trend towards lower RAO incidents in the “Less air” group, but the 95% confidence interval (CI) crosses the null effect line (CI = 0.78–1.19), indicating no statistically significant difference between the two groups. The weight of each study is proportional to its size, reflecting the individual contribution to the overall effect estimate, and the lack of heterogeneity (*I*^2^ = 0%) suggests consistency among the included studies.

#### 3.4.2 Sensitivity analysis of RAO incidence findings in TR band studies

**Figure 9:**
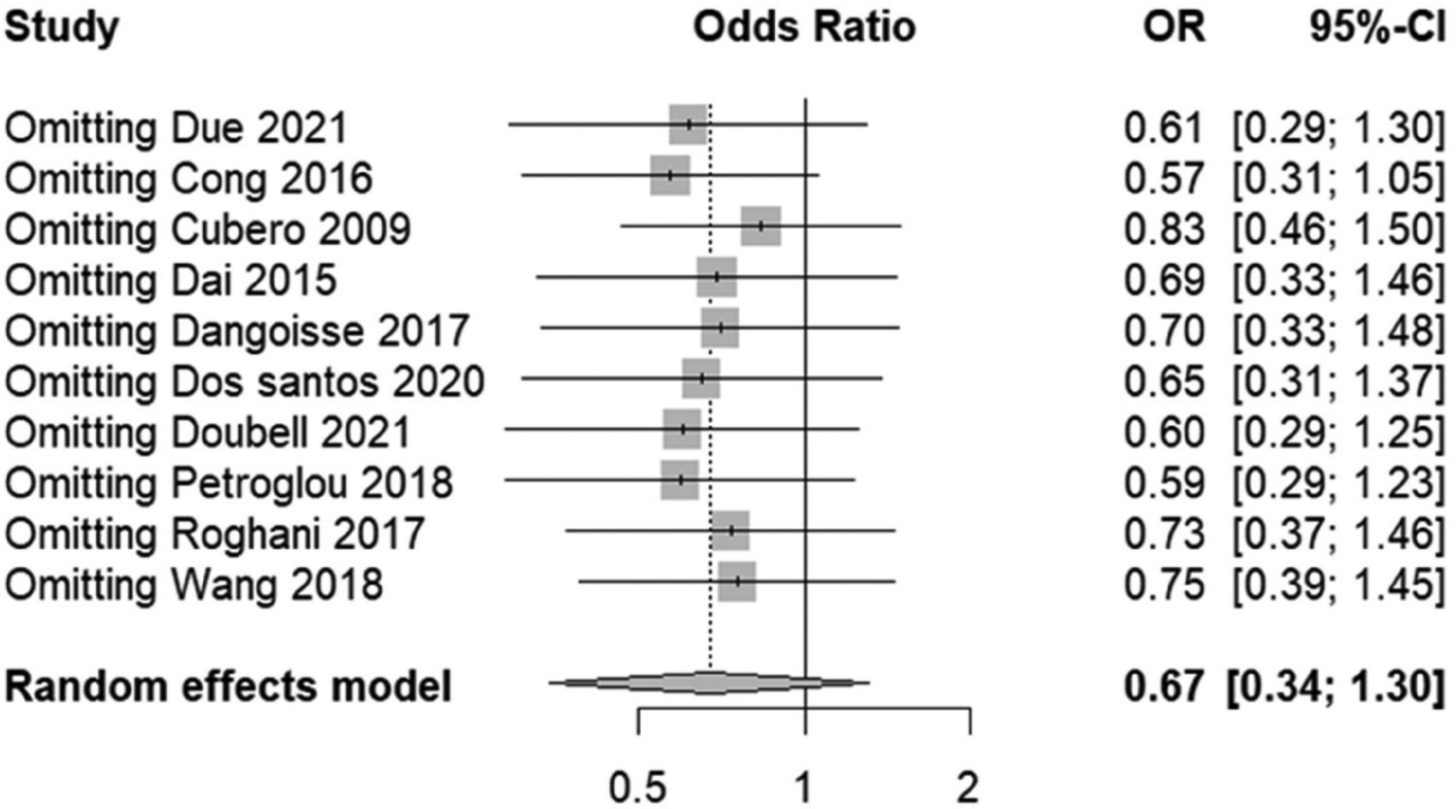
Sensitivity analysis of RAO incidence, reflecting stability upon the exclusion of individual studies.

In the sensitivity analysis plot (Figure 9), the central vertical line represents the null effect (OR = 1), where points to the left suggest a lower risk of RAO in the “Less air” group and points to the right suggest a higher risk. The plot exhibits minimal shifts in the combined OR when each study is systematically omitted, indicating that no individual study has a disproportionate influence on the meta-analysis outcome. The consistency of the recalculated ORs close to the overall OR (0.67) and within the original confidence interval supports the meta-analysis's conclusion that air volume does not significantly affect the risk of RAO in patients undergoing transradial band procedures.

**Figure 10:**
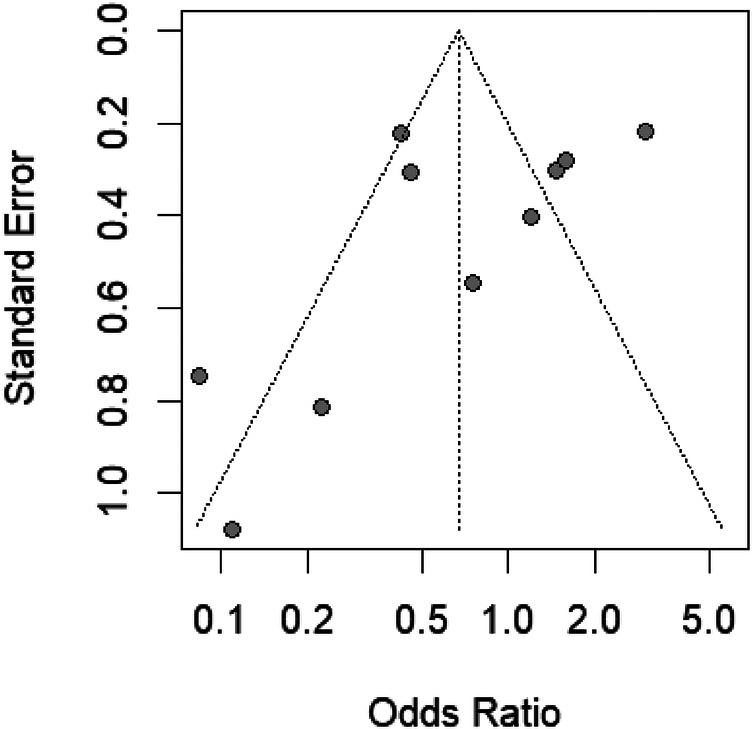
Funnel plot for assessing publication bias in RAO studies with transradial band devices, displaying symmetry.

The funnel plot's (Figure 10) symmetry around the pooled effect size suggests an unbiased distribution of study results, a visual indication corroborated by Egger's test, which shows no significant statistical evidence of bias (t = −1.73, df = 8, *p*-value = 0.1222). The Egger's test intercept also reinforces this finding, with a value close to zero and a nonsignificant *p*-value, indicating that the studies’ effect sizes are not disproportionately skewed by their standard errors. Together, these analyses suggest that our meta-analysis outcomes are likely free from the influence of publication bias, lending confidence to the overall conclusion that varying air volumes in TR bands do not significantly impact the incidence of RAO.

### 3.5 VAS score

#### 3.5.1 VAS score differences in TR band air volume interventions

**Figure 11:**
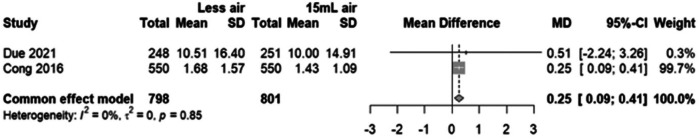
Forest plot demonstrating the mean difference in VAS scores between “Less air” and “15 ml air” TR band groups.

The forest plot (Figure 11) depicts a meta-analysis of mean differences in Visual Analogue Scale (VAS) scores between patients receiving “Less air” vs. “15 ml air” in transradial band devices (20, 23). The common effect model indicates a small but statistically significant higher mean VAS score in the “Less air” group [MD = 0.25, 95% CI (0.09, 0.41)], suggesting increased pain or discomfort. The minimal heterogeneity (*I*^2^ = 0%, *p* = 0.85) among the combined studies emphasizes consistency in the effect across different research settings. The calculated mean differences are robust, with the weight of the studies due to their sample sizes contributing significantly to the common effect model, affirming that a lesser air volume in the TR band may be associated with a small increase in reported pain.

#### 3.5.2 Analysis of VAS score variations by air volume in TR band devices

**Figure 12:**
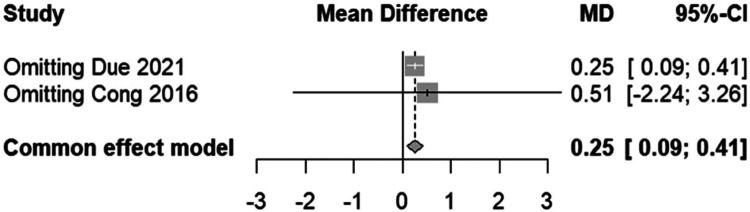
Sensitivity forest plot for VAS scores, highlighting the effect of omitting individual studies.

This sensitivity forest plot (Figure 12) illustrates the effect on the mean difference in Visual Analogue Scale (VAS) scores when specific studies are omitted from the meta-analysis. The common effect model shows a mean difference (MD) of 0.25 with a 95% confidence interval (CI) from 0.09 to 0.41, which remains consistent and statistically significant, suggesting that patients in the “Less air” group report higher pain scores than those in the “15 ml air” group. Omission of Due 2021 and Cong 2016 individually shows that neither study significantly alters this overall result, as indicated by the mean differences aligning closely with the combined estimate. This consistency in results, reflected in the narrow confidence intervals that do not cross the null effect line (MD = 0), reinforces the conclusion that the amount of air in the TR band is associated with a statistically significant difference in patient-reported pain levels.

### 3.6 Compression time

#### 3.6.1 Effect of TR band air volume on compression time

**Figure 13:**
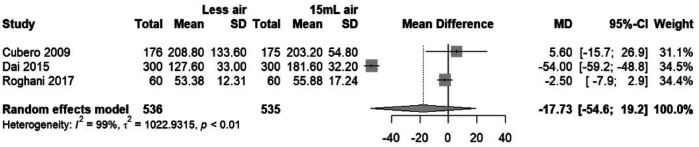
Forest plot of mean difference in compression time between “Less air” and “15 ml air” TR band groups.

The forest plot (Figure 13) indicates a mean difference in compression times between the “Less air” and “15 ml air” TR band groups. The random effects model shows a pooled mean difference of −17.73 with a 95% confidence interval of [−54.65; 19.20], which encompasses zero, suggesting that there is no statistically significant difference in compression times between the two groups. Despite this, there is considerable heterogeneity among the studies (*I*^2^ = 99%), indicating that the result should be interpreted with caution as the variation between studies is substantial.

#### 3.6.2 Sensitivity analysis of compression time findings across studies

**Figure 14:**
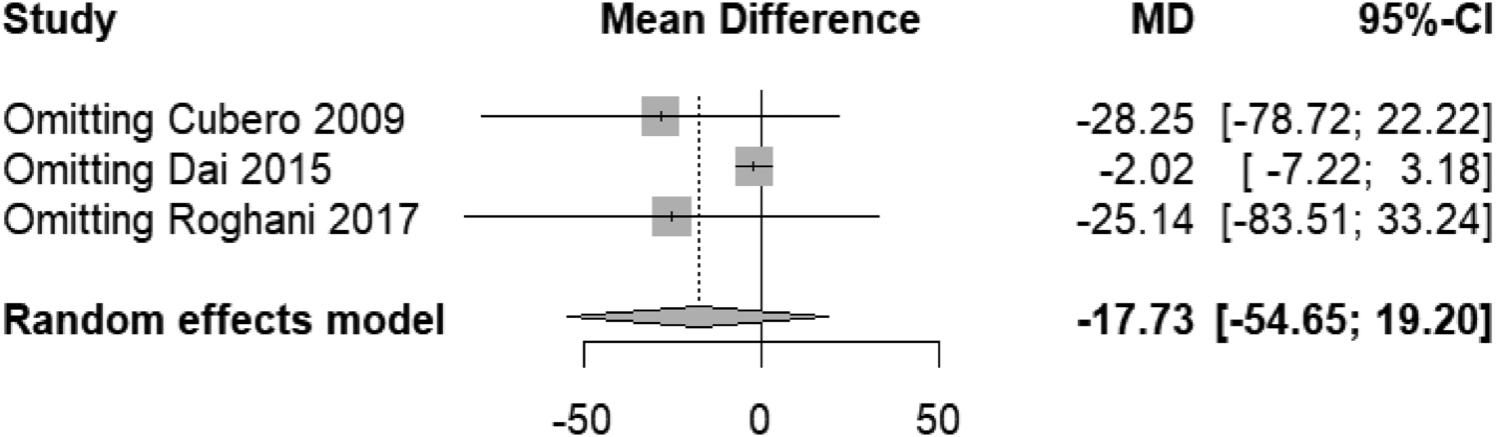
Sensitivity analysis showing the influence of individual study omission on compression time differences.

In the sensitivity analysis plot (Figure 14), the individual omission of studies from the meta-analysis does not significantly alter the overall mean difference estimate, which remains non-significant and crosses the null effect line. This suggests the meta-analysis findings regarding compression times are stable and not dependent on any single study. However, the wide confidence intervals imply considerable variability in the study results, which could be due to differences in study design, populations, or other factors not accounted for in the meta-analysis.

The analysis of compression time revealed substantial heterogeneity across the studies included in this meta-analysis. The variations in compression time could be attributed to multiple factors such as differences in patient characteristics (e.g., age, comorbidities), procedural techniques (e.g., catheterization duration, operator experience), and the methodological differences in how compression time was reported across studies. The inclusion of studies with a wide range of compression times, from short intervals to extended periods, may contribute to the inconsistency in outcomes observed. Although subgroup analysis was conducted to investigate the influence of these factors, the high variability necessitates cautious interpretation of the findings. Future studies that standardize compression protocols may help mitigate this issue and yield more reliable results.

## 4 Discussion

To compile a robust dataset for this meta-analysis, an extensive literature search was conducted across multiple databases, focusing on the efficacy of various interventions in preventing TRA complications. The databases searched included PubMed, Cochrane Library, and Embase, utilizing specific keywords and combinations such as “Transradial” and “Transradial AND pressure” or “Transradial AND blood”. This search strategy aimed to capture a broad spectrum of studies relevant to TRA complications and interventions, particularly focusing on the application pressures and decompression times associated with the TR Band. The search yielded a total of 3,508 papers from PubMed (with 2 duplicates), 825 trials from Cochrane (with 1 duplicate), and 1,364 papers from Embase (with 610 duplicates), indicating a significant interest and body of research in this area (PubMed, 2021; Cochrane, 2021; Embase, 2021). This comprehensive search underscores the extensive scientific effort dedicated to optimizing post-catheterization care and highlights the significant variability in approaches to managing transradial access (TRA) complications.

Our comprehensive analysis distilled insights from an initial pool of 5,697 papers, ultimately focusing on 11 pivotal studies. These studies, both observational and randomized by design, involved a total of 6,842 patients and provided a rich comparative lens on interventions using different air volumes in TR bands. The research efforts of Bardooli, Due, Cong, Cubero, Dai, Dangoisse, Dos Santos, Doubell, Kang, Petroglou, Roghani, and Wang were instrumental in offering a comprehensive dataset that spanned a wide range of air volume and pressure applications (11–24).

From our meta-analytical process, we identified no statistically significant difference in bleeding risks or hematoma formation between the groups assigned different air volumes, with an aggregate odds ratio (OR) for bleeding risk standing at 1.04 (95% CI: 0.60–1.82, *p* = 0.89) and for hematoma incidence at 0.96 (95% CI: 0.78–1.19, *p* = 0.95). This consistency across studies suggests that minor adjustments in air volume, within the studied ranges, do not materially affect these clinical outcomes, supporting a perspective of potential standardization in TR band application protocols that do not compromise patient safety or efficacy.

Moreover, the analysis presented no significant variance in the incidence of radial artery occlusion (RAO) across the different air volumes, with a pooled odds ratio of 0.96 (95% CI: 0.78–1.19, *p* = 0.95). This finding, reflective of the contributions from studies like those by Cubero, Lombardo (21) and Dai, Xu (11), reinforces the notion that air volume adjustments within TR bands do not substantially impact the risk of RAO post-catheterization, aligning with prior literature that underscores the potential for procedural standardization (26, 27).

Contrastingly, the meta-analysis unveiled a statistically significant difference in patient-reported pain levels, as measured by the Visual Analogue Scale (VAS), with the “Less air” group reporting higher levels of discomfort compared to the “15 ml air” group [Mean Difference (MD) = 0.25, 95% CI: 0.09–0.41, *p* < 0.01]. This distinction underscores the importance of balancing procedural efficacy with patient comfort, highlighting the critical role of optimizing air volume in TR bands for enhanced patient experiences without compromising clinical outcomes (28).

The examination of compression times, although not revealing a statistically significant difference between groups, highlighted pronounced heterogeneity (*I*^2^ = 99%). This outcome suggests that while air volume adjustments might not directly influence the duration necessary for effective hemostasis, there is a considerable variation in how compression times are impacted by other factors, necessitating further investigation.

In weaving together these findings, our meta-analysis challenges the previously held assumption that precise control over TR band air volume directly translates to improved clinical outcomes. Instead, it suggests a nuanced scenario where variations in air volume, within certain thresholds, do not significantly influence rates of bleeding, hematoma formation, or RAO but do have a marked effect on patient comfort. This revelation promotes a balanced approach to clinical practice, where safety and efficacy are prioritized alongside patient comfort. Furthermore, the observed heterogeneity and occasional lack of comparability among studies underscore the imperative for standardized methodologies in future research endeavors. By establishing uniform protocols for TR band application and outcome measurement, the applicability and clarity of research findings could be significantly enhanced, leading to the development of more precise, evidence-based clinical guidelines.

Ultimately, our meta-analysis advocates for a nuanced understanding of TR band application protocols, suggesting that within specific thresholds, air volume adjustments do not materially impact clinical safety outcomes but play a significant role in patient comfort. This insight emphasizes the need for future research to delineate clear guidelines that balance clinical innocuity with maximizing patient comfort, thereby contributing to the evolution of patient-centered post-catheterization care protocols.

## 5 Limitations and future directions

Despite the comprehensive nature of this meta-analysis, it is not without its limitations. One of the primary constraints lies in the inherent heterogeneity of the studies analyzed, which encompasses a wide array of interventions, methodologies, and patient populations. This diversity, while offering a broad perspective, also complicates the process of drawing definitive conclusions about the optimal air volume for TR bands. This meta-analysis is subject to several limitations, including the potential for publication bias. Studies with positive or significant results are more likely to be published, leading to an overrepresentation of favorable outcomes. This bias could potentially skew the overall findings of this meta-analysis, especially given that most studies included in the analysis reported favorable outcomes related to the use of TR Band in minimizing complications. Additionally, there was variability in the outcome measurements across the included studies. Some studies defined radial artery occlusion (RAO), hematoma, and patient discomfort using different criteria or scales, which could introduce inconsistencies in the assessment of the outcomes. The lack of standardized outcome measures across studies may undermine the ability to directly compare the efficacy of TR Band use in different clinical settings. Therefore, while our findings offer valuable insights, they should be interpreted with caution, and future studies should prioritize standardized outcome reporting to improve the reliability and comparability of results.

## 6 Clinical implications

Based on the findings of this meta-analysis, it is recommended that clinicians consider 15 ml of air volume as a standard for optimal use of the TR Band, as this volume provided a balance between hemostasis and patient comfort. However, it is essential to recognize that individual patient needs and preferences should be considered when determining the appropriate air volume, as patient comfort scores were notably higher in groups with reduced air volumes. Clinicians should also take into account the procedural context, such as catheterization duration and patient comorbidities, when selecting the appropriate compression time. While our findings do not indicate significant differences in bleeding risk or RAO between groups, optimizing the settings of the TR Band may enhance patient comfort without compromising safety. Further clinical studies with larger, more diverse populations are necessary to refine these recommendations and develop standardized protocols for TR Band use.

## 7 Conclusion

This meta-analysis presents a nuanced overview of the impact of transradial band air volume adjustments post-catheterization, highlighting that while such modifications do not significantly affect clinical outcomes such as bleeding, hematoma formation, and radial artery occlusion, they play a critical role in patient comfort. The findings challenge the prevailing notion of a linear relationship between air volume control and improved clinical outcomes, suggesting instead a plateau effect where variations within certain thresholds have minimal impact on safety outcomes but significantly influence patient experience. This insight underscores the importance of a balanced approach to post-catheterization care, advocating for protocols that optimize both procedural efficacy and patient comfort. Future research, through more rigorous and standardized methodologies, is essential to refine these insights and guide clinical practice towards enhanced patient-centered care.

## Data Availability

The datasets presented in this article are not readily available due to restrictions imposed by the hospital's regulations and policies. Requests to access the datasets should be directed to Zai-Guang Yao, u0nx3w@163.com.
