# Correction: Enhancing heart and circulatory well-being through optimized radial artery techniques: a meta-analysis of hemostasis and patient comfort

**DOI:** 10.3389/fcvm.2026.1797736

**Published:** 2026-02-11

**Authors:** Yanru Yang, Hongyan Zhu, Guangyao Zhai

**Affiliations:** Department of Cardiovascular Medicine, Capital Medical University, Beijing LuHe Hospital, Beijing, China

**Keywords:** radial artery hematoma radial artery compression time, radial artery occlusion, bleeding complications, patient comfort radial access, transradial band device

Author Guangyao Zhai was erroneously spelled as Guangyao Zai.

The original version of this article has been updated.

